# Supplementary material for: Identification of a Pathogenic Mutation for Glycogen Storage Disease Type II (Pompe Disease) in Japanese Quails (Coturnix japonica)
Source: Genes (Basel). 2025 Aug 19;16(8):975. doi: 10.3390/genes16080975 (PMC12386088; doi:10.3390/genes16080975)
Supplement: Supplementary file 1 [file genes-16-00975-s001.zip › Table_S1.pdf]

**Table S1.** Positions of all exons and introns in the *GAA I* gene of Japanese quails.

| Exon/Intron | Position (*Coturnix japonica* 2.1) | Length (base pairs) |
|---|---|---|
| Exon 1 | g.2688849_2689388 | 540 |
| Intron 1 | g.2688687_2688848 | 162 |
| Exon 2 | g.2688541_2688686 | 146 |
| Intron 2 | g.2687654_2688540 | 887 |
| Exon 3 | g.2687488_2687653 | 166 |
| Intron 3 | g.2687243_2687487 | 245 |
| Exon 4 | g.2687146_2687242 | 97 |
| Intron 4 | g.2686337_2687145 | 809 |
| Exon 5 | g.2686217_2686336 | 120 |
| Intron 5 | g.2686030_2686216 | 187 |
| Exon 6 | g.2685911_2686029 | 119 |
| Intron 6 | g.2685843_2685910 | 68 |
| Exon 7 | g.2685711_2685842 | 132 |
| Intron 7 | g.2684962_2685710 | 749 |
| Exon 8 | g.2684851_2684961 | 111 |
| Intron 8 | g.2684231_2684850 | 620 |
| Exon 9 | g.2684117_2684230 | 114 |
| Intron 9 | g.2683854_2684116 | 263 |
| Exon 10 | g.2683769_2683853 | 85 |
| Intron 10 | g.2683487_2683768 | 282 |
| Exon 11 | g.2683369_2683486 | 118 |
| Intron 11 | g.2683086_2683368 | 283 |
| Exon 12 | g.2682952_2683085 | 134 |
| Intron 12 | g.2682807_2682951 | 145 |
| Exon 13 | g.2682655_2682806 | 152 |
| Intron 13 | g.2682586_2682654 | 69 |
| Exon 14 | g.2682437_2682585 | 149 |
| Intron 14 | g.2682344_2682436 | 93 |
| Exon 15 | g.2682202_2682343 | 142 |
| Intron 15 | g.2682021_2682201 | 181 |
| Exon 16 | g.2681922_2682020 | 99 |
| Intron 16 | g.2681785_2681921 | 137 |
| Exon 17 | g.2681620_2681784 | 165 |
| Intron 17 | g.2681552_2681619 | 68 |
| Exon 18 | g.2681405_2681551 | 147 |
| Intron 18 | g.2681232_2681404 | 173 |
| Exon 19 | g.2681169_2681231 | 63 |

Positions were predicted based on the JQ whole genome shotgun sequence (NCBI reference sequence: NC_029529.1; *Coturnix japonica* 2.1) and JQ *GAA I* mRNA sequence (GenBank accession number: AB000967.1).
