# Supplementary material for: Identification of a Pathogenic Mutation for Glycogen Storage Disease Type II (Pompe Disease) in Japanese Quails (Coturnix japonica)
Source: Genes (Basel). 2025 Aug 19;16(8):975. doi: 10.3390/genes16080975 (PMC12386088; doi:10.3390/genes16080975)
Supplement: Supplementary file 1 [file genes-16-00975-s001.zip › Table_S2.pdf]

Table S2. Sequences of the primers and probes used in the Sanger sequencing and real-time polymerase chain reaction assay for the Japanese quail *GAA I* gene and c.1096delG mutation.

Primer/probe	Sequence 5′ to 3′ (mer)	Position (*Coturnix japonica* 2.1)	Tm (°C)	Ta (°C)	Amplicon size (bp)
Sanger sequencing:					
E1(1)-F	CCATAACCGTCCACGTTTCA (20)	g.2689419_2689438	65.55	57.0	211
E1(1)-R	AGCAGCACGGCAGTGATC (18)	g.2689228_2689245	65.52		
E1(2)-F	ATGCGACCACCCCGACA (17)	g.2689296_2689312	69.20	57.0	208
E1(2)-R	ACGTTCCTCTCGGGGTAG (18)	g.2689105_2689122	61.68		
E1(3)-F	ACAGTGCCACCTGGTCC (17)	g.2689145_2689161	62.41	57.0	212
E1(3)-R	TCATGCCCAGCGCTGTCT (18)	g.2688950_2688967	68.50		
E1(4)-F	CCTGGTGCTTCTACCCAC (18)	g.2689004_2689021	60.71	58.2	213
E1(4)-R	CAGGGAACGGTCACCTAC (18)	g.2688809_2688826	60.56		
E2-F	TTGTCCTCCTGCTTTGCTG (19)	g.2688718_2688736	64.18	58.0	219
E2-R	GCACAGAAGAGGTGGGCT (18)	g.2688518_2688535	63.14		
E3-F	CACAGTGAGGGGATGACC (18)	g.2687694_2687711	61.67	57.0	242
E3-R	TCCCTGCCCCAGTGGTA (17)	g.2687470_2687486	65.43		
E4(1)-F	TAGCCCAAGTCTCACGGC (18)	g.2687302_2687319	64.06	58.2	173
E4(1)-R	CATGGCATTGCTGTTGAGGA (20)	g.2687147_2687166	67.19		
E4(2)-F	CAACCTGTACGGCGCTCA (18)	g.2687217_2687234	66.21	57.0	169
E4(2)-R	AACGGAGCCATCGGACCA (18)	g.2687066_2687083	69.13		
E5-F	ATGCTCCCCAGGTCTCAC (18)	g.2686363_2686380	62.83	57.0	195
E5-R	GGGAAGAGCACTTTATGGTG (20)	g.2686186_2686205	61.06		
E6-F	CTCTCCCATCCCTTTGCC (18)	g.2686060_2686077	64.59	57.0	213
E6-R	AGGCAGCAGCACACAGC (17)	g.2685865_2685881	64.39		
E7-F	ATGGAAGGAGGGAGGGCT (18)	g.2685886_2685903	64.90	58.0	226
E7-R	AGTGACAGCCCTGAGCGT (18)	g.2685678_2685695	63.85		
E8-F	CTGAATCACACTGGCATTGC (20)	g.2685007_2685026	64.35	57.0	212
E8-R	CCTTTAAAGCCCCCAGGG (18)	g.2684815_2684832	65.36		
E9-F	TAGTGTTCTGCCCTGCTGAA (20)	g.2684256_2684275	63.54	57.0	196
E9-R	AGCAGGGAGATGGCACCA (18)	g.2684080_2684097	67.13		
E10-F	AGGCGTCACTAATCCCCC (18)	g.2683890_2683907	63.84	58.2	170
E10-R	AGCGCAGCCTGCATCTCA (18)	g.2683738_2683755	68.74		
E11-F	GTGTCCCTCACTCTCTCC (18)	g.2683521_2683538	56.95	58.2	221
E11-R	ATGCCTGAGCAGCACGGT (18)	g.2683318_2683335	67.27		
E12-F	TCCTCAGGGCTGCTCTCA (18)	g.2683117_2683134	65.19	57.0	246
E12-R	AAATCCCGACAGGGCTGG (18)	g.2682889_2682906	67.30		
E13-F	AGGCCACGAAACTCCCCA (18)	g.2682842_2682859	68.01	58.2	251
E13-R	CAACTGCACCATCCCCAC (18)	g.2682609_2682626	65.33		
E14-F	TGGAGATGTGCGTGGGGA (18)	g.2682620_2682637	69.29	58.2	256
E14-R	TTCCACCACCCGACCACA (18)	g.2682382_2682399	69.00		
E15-F	TGTGGTCGGGTGGTGGAA (18)	g.2682382_2682399	69.00	57.0	253
E15-R	AGAGCCTCATGCAAACCATC (20)	g.2682147_2682166	64.12		
E16-F	ACATGCTTGAGCCCATCACT (20)	g.2682059_2682078	64.66	57.0	204
E16-R	CCTCTGAGACTGTCCCCA (18)	g.2681875_2681892	61.52		
E17-F	TATCGTAGCCCTGCCCAC (18)	g.2681828_2681845	63.65	58.2	265
E17-R	TGTCCCATACACCCCCCT (18)	g.2681581_2681598	65.15		
E18-F	AGGGGGGTGTATGGGACA (18)	g.2681581_2681598	65.15	58.2	235
E18-R	TGGGCACCCTTAGGACAG (18)	g.2681364_2681381	63.76		
E19-F	GCCATATGTAGGGTTGAGGT (20)	g.2681286_2681305	60.23	57.0	188
E19-R	AACGTGCACATTGGGTCATG (20)	g.2681118_2681137	66.84		
Real time-polymerase chain reaction:					
Forward primer	TGGGCACACTGCAGGTTT (18)	g.2686026_2686043	65.59	60.0	67
Reverse primer	CCAACGGCAGAGGTGGAA (18)	g.2685977_2685994	67.42		
Probe (wild-type)	CCCTCTGGGCACTTG (15)	g.2685997_2686011	58.62		
Probe (mutant)	CCCCTCTGGCACTTG (15)	g.2685997_2686011	58.62		

Tm, melting temperature calculated using OlvTools (https://olvtools.com/tmvalue); Ta, annealing temperature used in this study. The positions of each pair of primers and probes designed in this study were based on the whole genome shotgun sequence (NCBI reference sequence: NC_029529.1; *Coturnix japonica* 2.1).
